# Predictive value of inflammation-based Glasgow prognostic score, platelet-lymphocyte ratio, and global registry of acute coronary events score for major cardiovascular and cerebrovascular events during hospitalization in patients with acute myocardial infarction

**DOI:** 10.18632/aging.203273

**Published:** 2021-07-16

**Authors:** Xiaoqun Xu, Long Cai, Tielong Chen, Shibiao Ding, Fengwei Zhang, Beibei Gao, Houyong Zhu, Jinyu Huang

**Affiliations:** 1 Centre of Laboratory Medicine, Affiliated Hangzhou Chest Hospital, Zhejiang University School of Medicine, Hangzhou, Zhejiang, China; 2 Department of Cardiology, Hangzhou TCM Hospital Affiliated to Zhejiang Chinese Medical University, Hangzhou, Zhejiang, China; 3 Department of Cardiology, The Affiliated Hangzhou First People’s Hospital, Zhejiang University School of Medicine, Hangzhou, Zhejiang, China

**Keywords:** acute myocardial infarction, major adverse cardiovascular and cerebrovascular events, inflammation-based Glasgow prognostic scores, platelet-lymphocyte ratio, global registry of acute coronary events

## Abstract

Purpose: The goal of this study was to evaluate the predictive ability of the inflammation-based Glasgow Prognostic Score (GPS), platelet-lymphocyte ratio (PLR), Global Registry of Acute Coronary Events (GRACE) score and combined diagnostic models for the occurrence of major adverse cardiovascular and cerebrovascular events (MACEs) in patients with acute myocardial infarction (AMI).

Methods: In this retrospective cohort study, eligible patients were required to meet the third global definition of myocardial infarction. The primary outcome of this study was the occurrence of MACEs during hospitalization. Receiver operating characteristic (ROC) curve analysis was performed to assess the predictive ability of the GPS, PLR, GRACE scores, and joint diagnostic models for primary outcomes; univariate and multivariate logistic regression analyses were performed.

Findings: A total of 175 patients were enrolled. The results of the univariate ROC curve analysis for the incidence of MACEs during hospitalization showed that the area under the curve (AUC) was 0.780 (95% confidence interval (CI) 0.696-0.864) for the GPS, 0.766 (95% CI 0.682-0.850) for the redefined GPS (RGPS), 0.561 (95% CI 0.458-0.664) for the PLR score (PLRS), and 0.793 (95% CI 0.706-0.880) for GRACE. Multivariate ROC curve analysis showed that the AUC value was 0.809 (95% CI 0.726-0.893) for the GPS combined with GRACE, 0.783 (95% CI 0.701-0.864) for the GPS combined with the PLRS, 0.794 (95% CI 0.707-0.880) for GRACE combined with the PLRS, and 0.841 (95% CI 0.761-0.921) for the GPS combined with GRACE and the PLRS. The combined diagnostic model including the GPS plus GRACE and the PLRS had a higher AUC value than the GPS, RGPS and GRACE models (P = 0.014, P = 0.004, and P = 0.038, respectively). The multivariate logistic regression model showed that the odds ratio for hospitalized MACEs was 5.573 (95% CI 1.588-19.554) for GPS scores of 2 versus 0, and the GRACE score was also an independent risk factor for MACEs, with an odds ratio of 1.023 (95% CI 1.009-1.036). Implications: The diagnostic model combining the GPS plus GRACE and the PLRS has better predictive ability for the occurrence of MACEs during hospitalization than each single score. Thus, the use of a combined GPS plus GRACE and PLRS model will be of clinical benefit in a broad group of individuals with AMI.

## INTRODUCTION

Acute myocardial infarction (AMI) is the leading cause of death worldwide, and the major adverse cardiovascular and cerebrovascular events (MACEs) resulting from AMI are not only a direct cause of death but also a huge economic burden on the health care system [1–3]. The American College of Cardiology (ACC)/American Heart Association (AHA) and the European Society of Cardiology (ESC) guidelines recommend the Global Registry of Acute Coronary Events (GRACE) score as one of the main tools for the risk assessment of patients with acute coronary syndrome (ACS) [4, 5]. It has a certain predictive ability for hospitalization and provides long-term mortality risk for patients with ACS [6], but its composition lacks inflammation assessment. Our previous study [7] showed that the inflammation-based Glasgow Prognostic Score (GPS) was similar to GRACE in predicting MACEs in patients with AMI. The combined model of the two scores may improve the predictive power for MACEs in patients with AMI. The GPS is composed of hypersensitive C-reactive protein (H-CRP) and albumin, and H-CRP is widely recognized as one of the strongest risk indicators for predicting cardiovascular disease in addition to being considered an important marker of inflammatory factors [8]. Albumin is considered to be as important as H-CRP in predicting the prognosis of myocardial infarction [9]. The reason may be that under stress, albumin serves as an important energy source, and its reduction indicates the energy loss of AMI patients. In addition, albumin has also been found to act against oxidative stress damage [10], which may be involved in the inhibition of platelet activation and anti-vascular endothelial cell apoptosis [11]. However, the GPS was derived from the prognostic data from cancer patients, and the cutoff values established based on their H-CRP and albumin levels may not be the most appropriate for patients with AMI. In addition, in recent years, some studies [12–14] have reported that the platelet-to-lymphocyte ratio (PLR) can also predict MACEs in ACS patients. Sia et al. [15] investigated AMI patients without undergoing percutaneous coronary intervention (PCI) and found that PLR was an independent predictor of thrombosis. Toprak et al. [16] found that a high level of PLR was associated with the no reflow phenomenon in ST-elevation myocardial infarction (STEMI) patients with undergoing primary PCI.

Therefore, in this study, we conducted a retrospective cohort study to jointly evaluate the predictive ability of the GPS, PLR and GRACE in predicting adverse outcomes in patients with AMI during hospitalization and to optimize the calculation of the GPS.

## MATERIALS AND METHODS

### Participants

Patients were recruited from the Zhejiang Hospital of Integrated Traditional Chinese and Western Medicine from January 1, 2015, to October 9, 2020. An exemption from the informed consent requirement was approved by the ethics committee of Zhejiang Hospital of Integrated Traditional Chinese and Western Medicine (Ethical Application Ref: 2020KS195), as this was a retrospective cohort study. This study protocol strictly complied with the requirements of the Helsinki Declaration of the World Medical Association and the international ethics guide for human biomedical research of the Council for International Organizations of Medical Sciences (CIOMS).

### Study design

According to the GPS data from our previous study^7^, the optimal threshold was obtained by the Youden index, and the sensitivity of the optimal expectation was 0.883, and the specificity was 0.664. So the estimation of the sample size for evaluation of the diagnostic test was calculated [17, 18]; a sample size of at least 175 was calculated using MedSci Sample Size tools (MSST) (https://www.medsci.cn/medsci-tools).

According to the third global definition of myocardial infarction [19], 175 patients met the inclusion criteria during the retrospective retrieval period. The following exclusion criteria were used: (1) lack of data on H-CRP serum albumin, platelets or lymphocytes; and (2) critical patients discharged automatically without MACEs.

### Data collection

This process was similar to previous studies; in brief, the baseline data included age, sex, diagnosis, hypertension, diabetes, acute infection, autoimmune diseases, tumors, nephrotic syndrome, cirrhosis, uremia, MACEs, blood pressure, PCI type, Killip classification, and biochemical indicators, including H-CRP, albumin, platelets, lymphocytes, hemoglobin, creatine kinase (CK), creatine kinase MB (CK-MB), troponin I (TNI), type B natriuretic peptide (BNP), low-density lipoprotein (LDL), creatinine, D-dimer, and alanine aminotransferase (ALT). All biochemical indicators were selected as the first biochemical results after admission.

### Definition of each score

GPS was defined as follows: patients with an elevated H-CRP level (> 10 mg/L) and a low albumin level (< 35 g/L) were designated as a GPS of 2. The presence of one abnormality associated with either the H-CRP level or albumin level was designated as a GPS of 1. If both metrics were normal, its value was designated as 0 point.

The redefined GPS (RGPS) was based on high-sensitivity H-CRP and albumin data from the previous study^7^ and redefined by optimal thresholds: patients with an elevated H-CRP level (> 12.57 mg/L) and a low albumin level (< 35.95 g/L) were designated as a GPS of 2. The presence of one abnormality associated with either the H-CRP level or albumin level was designated as a GPS of 1. If both metrics were normal, its value was designated as 0 point.

The definition of the PLR score (PLRS) was based on the quartile range of the PLR in the previous study, as follows: patients with an elevated PLR (> 211.25) were designated as a PLRS of 2, but if patients with a decreased PLR (< 105.71) were designated as a PLRS of 0, its value was designated as 1 point (i.e., between the two).

According to Granger CB et al. [20–22], the GRACE score was completed. In brief, age, heart rate, systolic blood pressure, creatinine, prehospital cardiac arrest, Killip classification, ST segment deviation, and myocardial enzyme elevation were used to determine the GRACE score.

### End points

The primary outcome was MACEs during hospitalization, which were calculated as a combination of deterioration of heart failure, cardiogenic shock, cardiovascular death, mechanical complications of myocardial infarction, stroke, and persistent ventricular arrhythmias. For all eligible individuals, observation period from inclusion to the first occurrence of MACEs (Supplementary Figure 1).

### Statistical analysis

Data analysis referred to previous studies^7^. Briefly, continuous variables were summarized as medians and quartiles and compared using the Kruskal-Wallis test. Univariate/multivariate associations between clinical variables and the primary outcome were estimated by logistic regression analysis using a forward stepwise logistic regression (LR) model. The variables of clinical interest and clinical variables in the univariate analysis (P < 0.10) were included in multivariate analysis, but those variables that were included in the scoring system were excluded. The calibration of the multivariate logistic regression model was done using the Hosmer-Lemeshow good of fit test. The area under the curve (AUC) was calculated by receiver operating characteristic (ROC) curve analysis to determine the prediction of each score for the primary outcome. Pairwise comparisons of ROC curves were quantitatively analyzed by the Delong method. Subgroup analysis were completed according to the type of PCI, the type of AMI, and the presence or absence of acute infection. Expectation maximization (EM) was used to fill in missing variables. In addition, the optimal thresholds for the RGPS and PLRS were determined by comprehensive evaluation of the Youden, Product and Euclidean indices. The odds ratio (OR) and 95% confidence interval (CI), AUC and 95% CI, rate, and median (quartile) were calculated a summary statistic. The data were analyzed using SPSS 25.0 (SPSS, Inc., Chicago, IL).

## RESULTS

### Characteristics of the included patients

**Figure 1:**
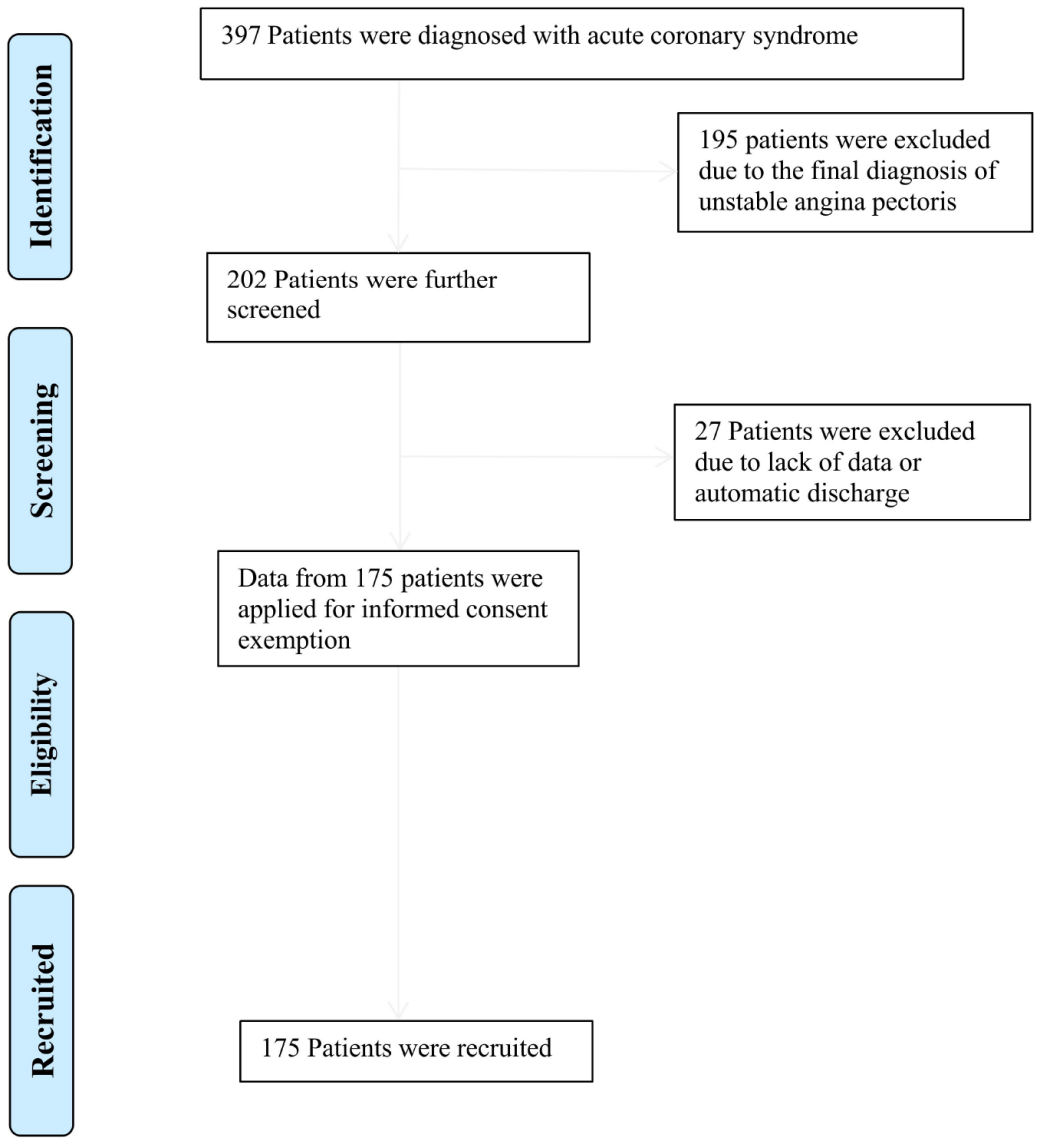
Flow diagram for recruitment of patients.

The main clinical features of the patients are shown in Table 1. A total of 175 patients were included in this study (Figure 1). The average age was 65 (54-80) years, 73.1% of patients were male, 60.6% of patients had a history of hypertension, 37.7% of patients had a history of diabetes mellitus, and 34.3% had acute infection. Of these patients, 40 (22.9%) had MACEs. The baseline characteristics of patients were categorized according to the GPSs. Compared with the low-score group, the high-score group had older patients (P < 0.001), a higher prevalence of diabetes (P < 0.05), a higher acute infection rate (P < 0.001), a higher prevalence of severe kidney disease (P = 0.015), lower hemoglobin levels (P < 0.001), higher Killip classes (P < 0.001), lower blood lymphocyte levels (P<0.001), higher D-dimer levels (P < 0.001), higher creatinine levels (P < 0.001) and higher BNP levels (P < 0.001). There were missing values for LDL, BNP, and D-dimers, with missing rates of 1.7%, 3.4, and 1.7, respectively, that were completed through the EM method (Supplementary Table 1).

**Table 1:** Relationships between clinical characteristics and the GPS in patients with acute myocardial infarction.

**Variable**	**GPS**			***P***
	**0**	**1**	**2**	
Age, (years)	58.0(49.0-70.5)	66.0(58.0-81.0)	80.0(63.5-89.0)	<0.001
Males, (n, %)	57(78.1)	42(70.0)	29(69.0)	0.457
Hypertension, (n, %)	44(60.3)	35(58.3)	27(64.3)	0.831
Diabetics, (n, %)	21(28.8)	25(41.7)	20(47.6)	0.098
Acute infections, (n, %)	9(12.3)	23(38.3)	28(66.7)	<0.001
Autoimmune diseases, (n, %)	1(1.4)	3(5.0)	3(7.1)	0.225
Nephrotic syndrome or uremia, (n, %)	0(0)	2(3.3)	4(9.5)	0.015
Liver cirrhosis, (n, %)	0(0)	1(1.7)	2(4.8)	0.111
Heart rate, (times/min)	78.0(68.0-89.5)	76.5(70.0-99.8)	86.0(75.0-96.0)	0.066
SBP, (mmHg)	130.0(114.0-146.5)	133.0(120.0-146.0)	128.5(108.0-150.0)	0.553
DBP, (mmHg)	74.0(67.5-81.0)	72.0(68.0-88.8)	70.0(62.0-80.0)	0.200
Killip > 2, (n, %)	7(9.6)	19(31.7)	21(50.0)	<0.001
Hemoglobin, (g/L)	136.0(121.5-144.0)	122.0(110.0-141.0)	123.0(106.8-131.3)	<0.001
Platelet, (×10^12/L)	192.0(150.0-230.0)	191.5(153.0-235.5)	171.5(141.0-226.0)	0.788
Lymphocyte, (×10^12/L)	1.4(1.1-1.8)	1.4(1.0-1.8)	1.0(0.7-1.4)	<0.001
D-Dimer, (mg/L)	0.3(0.2-0.5)	0.6(0.3-1.2)	1.5(0.7-2.9)	<0.001
ALT, (U/L)	29.0(18.5-53.0)	36.0(22.0-55.0)	28.5(17.0-53.3)	0.465
CK, (U/L)	462.0(163.5-1734.5)	866.0(176.8-1652.8)	261.5(96.8-622.3)	0.019
CK-MB, (U/L)	51.0(17.5-157.0)	59.0(20.0-126.0)	28.5(15.0-46.0)	0.031
LDL, (mmol/L)	2.4(1.8-3.0)	2.2(1.6-2.6)	2.2(1.7-2.5)	0.335
Creatinine, (umol/L)	87.6(76.4-97.8)	90.9(81.4-5101.1)	112.2(85.3-127.3)	0.001
BNP, (pg/mL)	125.0(58.5-274.0)	380.0(145.8-688.3)	900.0(339.8-1634.3)	<0.001
TNI, (ng/mL)	9.8(2.3-48.7)	19.1(3.9-51.5)	8.1(1.3-28.0)	0.067

BNP, type B natriuretic peptide; CK, creatine kinase; CK-MB, creatine kinase MB; DBP, diastolic blood pressure; GPS, inflammation-based Glasgow Prognostic Score; GRACE, Global Registry of Acute Coronary Events; LDL, low density lipoproteincreatinine; PCI, percutaneous coronary intervention; SBP, systolic blood pressure; SD, standard deviation; TNI, troponin I. Values are numbers (%) or medians (interquartile ranges).

### Prediction of the primary outcome

**Figure 2:**
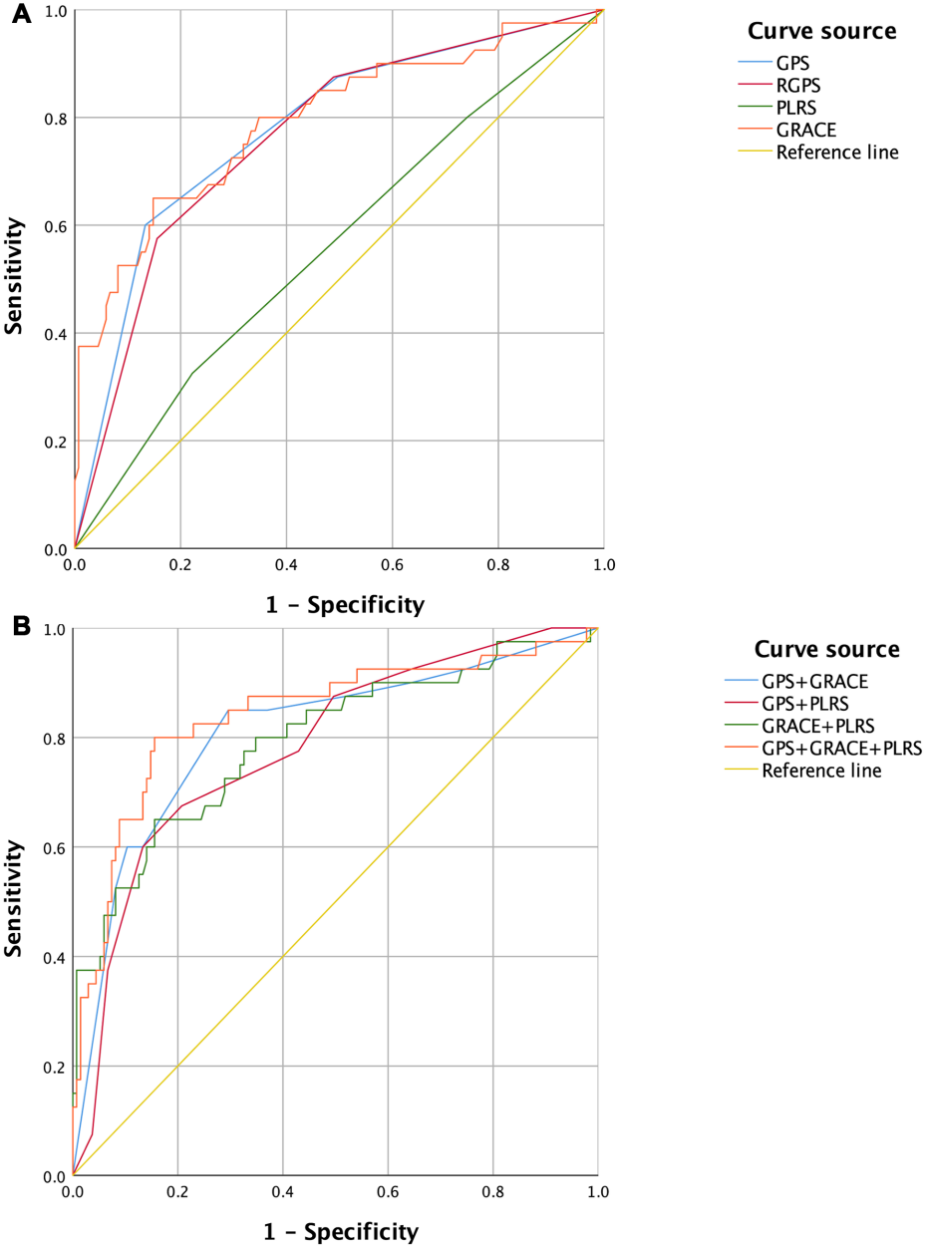
**ROC Curves of GPSs, PLRS, GRACE and combined diagnostic models for the occurrence of MACEs in patients with acute myocardial infarction.** (**A**) Univariate ROC curves. (**B**) Multivariate ROC curves. GPS, inflammation-based Glasgow Prognostic Score; GRACE, Global Registry of Acute Coronary Events; MACEs, major adverse cardiovascular and cerebrovascular events; PLRS, platelet-to-lymphocyte score; RGPS, Redefined inflammation-based Glasgow Prognostic Score; ROC, receiver operating characteristic.

The results of unit ROC curve analysis for the MACEs showed that the AUCs were 0.780 (95% CI 0.696-0.864) for the GPS, 0.766 (95% CI 0.682-0.850) for RGPS, 0.561 (95% CI 0.458-0.664) for PLRS, and 0.793 (95% CI 0.706-0.880) for GRACE (Figure 2A and Table 2). The GPS, RGPS and GRACE had higher AUC values than the PLRS (P < 0.001, P < 0.001, and P < 0.001, respectively) (Supplementary Table 2).

Multivariate ROC curve analysis revealed that this AUC value was 0.809 (95% CI 0.726-0.893) for the GPS combined with GRACE, 0.783 (95% CI 0.701-0.864) for the GPS combined with the PLRS, 0.794 (95% CI 0.707-0.880) for GRACE combined with the PLRS, and 0.841 (95% CI 0.761-0.921) for the GPS combined with GRACE and the PLRS (Figure 2B and Table 2 and Supplementary Table 3). The combined diagnostic model of the GPS plus GRACE and the PLRS had a higher AUC value than the combination of the GPS, RGPS and GRACE (P = 0.014, P = 0.004, and P = 0.038, respectively) (Supplementary Table 4).

**Table 2:** ROC analysis of in-hospital MACEs.

**Variable**	**AUC**	**CI**	**P**
GPS	0.780	0.696-0.864	<0.001
RGPS	0.766	0.682-0.850	<0.001
PLRS	0.561	0.458-0.664	0.241
GRACE	0.793	0.706-0.880	<0.001
GPS+GRACE	0.809	0.726-0.893	<0.001
GPS+PLRS	0.783	0.701-0.864	<0.001
GRACE+PLRS	0.794	0.707-0.880	<0.001
GPS+GRACE+PLRS	0.841	0.761-0.921	<0.001

AUC, area under the curve; CI, confidence interval; GPS, inflammation-based Glasgow Prognostic Score; GRACE, Global Registry of Acute Coronary Events; MACEs, Global Registry of Acute Coronary Events; PLRS, platelet-to-lymphocyte score; RGPS, Redefined inflammation-based Glasgow Prognostic Score.

### Logistic regression analysis

Univariate logistic regression analysis revealed that the OR for in-hospital MACEs was 3.053 (95% CI 0.997-9.349) for the GPS (1 vs 0) (Table 3), 18.133 (95% CI 0.997-9.349) for the GPS (2 vs 0), 1.029 (95% CI 1.018-1.039) for the GRACE score, 1.052 (95% CI 1.024-1.081) for age, 5.517 (95% CI 2.588-11.762) for acute infection, 7.389 (95% CI 1.301-41.965) for severe renal damage, 0.515 (95% CI 0.282-0.941) for PCI type, 1.027 (95% CI 1.005-1.049) for heart rate, 2.850 (95% CI 1.964-4.136) for Killip classification, 0.963 (95% CI 0.945-0.982) for hemoglobin, 0.411 (95% CI 0.207-0.817) for lymphocyte count, 1.604 (95% CI 1.214-2.121) for D-dimer, 0.676 (95% CI 0.592-0.771) for albumin, 1.011 (95% CI 1.002-1.019) for H-CRP, 1.012 (95% CI 1.003-1.022) for creatinine and 1.001 (95% CI 1.001-1.002) for BNP.

**Table 3:** Logistic regression analysis of MACEs during hospitalization.

**Variable**	**OR**	**Univariate analysis for 95% CI**	***P***	**OR**	**Multivariate analysis for 95% CI**	***P***
GPS			<0.001			0.016
GPS (1 vs 0)	3.053	0.997-9.349	0.051	1.810	0.536-6.113	0.339
GPS (2 vs 0)	18.133	6.068-54.185	<0.001	5.573	1.588-19.554	0.007
PLRS	1.395	0.837-2.323	0.201			0.326
GRACE score	1.029	1.018-1.039	<0.001	1.023	1.009-1.036	0.001
Age	1.052	1.024-1.081	<0.001			-
Males	0.518	0.244-1.100	0.087			0.742
Hypertension	1.277	0.612-2.662	0.515			0.839
Diabetics	1.694	0.829-3.461	0.148			0.445
Acute infections	5.517	2.588-11.762	<0.001			0.136
Autoimmune diseases	2.655	0.569-12.396	0.214			-
Nephrotic syndrome or uremia	7.389	1.301-41.965	0.024			0.740
Liver cirrhosis	7.053	0.623-79.897	0.115			-
PCI type	0.515	0.282-0.941	0.031			0.332
Heart rate	1.027	1.005-1.049	0.015			-
SBP	1.000	0.985-1.016	0.976			-
DBP	0.988	0.962-1.014	0.362			-
Killip class	2.850	1.964-4.136	<0.001			-
Hemoglobin	0.963	0.945-0.982	<0.001			0.091
Platelet	0.998	0.993-1.003	0.456			-
Lymphocyte	0.411	0.207-0.817	0.011			-
D-dimer	1.604	1.214-2.121	0.001	1.208	0.993-1.471	0.059
ALT	1.006	0.999-1.014	0.086	1.013	0.999-1.026	0.060
CK	1.000	1.000-1.000	0.259			0.724
CK-MB	0.999	0.996-1.002	0.517			0.691
Albumin	0.676	0.592-0.771	<0.001			-
HS-CRP	1.011	1.002-1.019	0.011			-
LDL	0.678	0.430-1.070	0.095			0.407
Creatinine	1.012	1.003-1.022	0.013			0.439
BNP	1.001	1.001-1.002	<0.001			0.374
TNI	0.999	0.996-1.002	0.423			0.936

ALT, Alanine aminotransferase; BNP, type B natriuretic peptide; CI, confidence interval; CK, creatine kinase; CK-MB, creatine kinase MB; DBP, diastolic blood pressure; GPS, inflammation-based Glasgow Prognostic Score; GRACE, Global Registry of Acute Coronary Events; LDL, low density lipoproteincreatinine; MACEs, Global Registry of Acute Coronary Events; OR, odds ratio; PCI, percutaneous coronary intervention; PLRS, platelet-to-lymphocyte score; SBP, systolic blood pressure; TNI, troponin I.

The multivariate logistic regression model goodness-of-fit test was completed using the Hosmer-Lemeshow method, which showed that the model had sufficient calibration (P = 0.838). The results showed that the OR for hospitalized MACEs was 5.573 (95% CI 1.588-19.554) for GPS (2 vs 0), and the OR for MACEs was 1.023 (95% CI 1.009-1.036) for GRACE score (Table 3).

### Subgroup analysis

In the STEMI group, the AUC for in-hospital MACEs was 0.737 (95% CI 0.647-0.814) for the GPS, 0.732 (95% CI 0.643-0.810) for the RGPS, 0.586 (95% CI 0.491-0.676) for the PLRS, and 0.788 (95% CI 0.703-0.858) for GRACE (Supplementary Figure 2 and Supplementary Table 5). The AUC was 0.807 (95% CI 0.724-0.874) for the GPS combined with GRACE, 0.738 (95% CI 0.649-0.815) for the GPS combined with the PLRS, 0.791 (95% CI 0.706-0.860) for GRACE combined with the PLRS, and 0.816 (95% CI 0.734-0.882) for the GPS combined with GRACE and the PLRS. The combined diagnostic model of the GPS plus GRACE and the PLRS had a higher AUC value than the GPS and RGPS (P = 0.0274 and P = 0.0231, respectively). In the non-ST-elevation myocardial infarction (NSTEMI) group, the AUCs were 0.864 (95% CI 0.749-0.940) for the GPS, 0.830 (95% CI 0.708-0.916) for the RGPS, 0.511 (95% CI 0.376-0.644) for the PLRS, and 0.877 (95% CI 0.764-0.948) for GRACE. The AUC was 0.913 (95% CI 0.809-0.971) for the GPS combined with GRACE, 0.869 (95% CI 0.755-0.943) for the GPS combined with the PLRS, 0.877 (95% CI 0.764-0.948) for GRACE combined with the PLRS, and 0.912 (95% CI 0.808-0.971) for the GPS combined with GRACE and the PLRS. The combined diagnostic model of the GPS plus GRACE seemed to have a higher AUC value than each single score, but there was no significant difference. The combined diagnostic model of the GPS plus GRACE and the PLRS had a higher AUC value than the RGPS (P = 0.0483).

In the PCI group, the AUC for in-hospital MACEs was 0.738 (95% CI 0.654-0.811) for the GPS, 0.731 (95% CI 0.647-0.805) for the RGPS, 0.522 (95% CI 0.433-0.610) for the PLRS, and 0.704 (95% CI 0.618-0.781) for GRACE (Supplementary Figure 3 and Supplementary Table 6). The AUC was 0.760 (95% CI 0.677-0.830) for the GPS combined with GRACE, 0.763 (95% CI 0.681-0.833) for the GPS combined with the PLRS, 0.718 (95% CI 0.633-0.793) for GRACE combined with the PLRS, and 0.776 (95% CI 0.695-0.844) for the GPS combined with GRACE and the PLRS. The combined diagnostic model of the GPS plus GRACE and the PLRS seemed to have a higher AUC value than each single score, but there was not statistically significantly different. In the non-PCI group, the AUCs were 0.808 (95% CI 0.662-0.911) for the GPS, 0.788 (95% CI 0.638-0.896) for the RGPS, 0.642 (95% CI 0.483-0.780) for the PLRS, and 0.828 (95% CI 0.684-0.925) for GRACE. This AUC was 0.856 (95% CI 0.718-0.944) for the GPS combined with GRACE, 0.826 (95% CI 0.682-0.924) for the GPS combined with the PLRS, 0.834 (95% CI 0.692-0.929) for GRACE combined with the PLRS, and 0.862 (95% CI 0.725-0.948) for the GPS combined with GRACE and the PLRS. The combined diagnostic model of the GPS plus GRACE and the PLRS had a higher AUC value than the RGPS alone (P = 0.0429).

In the acute infection group, the AUC for in-hospital MACEs was 0.693 (95% CI 0.560-0.806) for the GPS, 0.664 (95% CI 0.530-0.781) for the RGPS, 0.551 (95% CI 0.417-0.680) for the PLRS, and 0.700 (95% CI 0.568-0.812) for GRACE (Supplementary Figure 4 and Supplementary Table 7). This AUC was 0.762 (95% CI 0.635-0.863) for the GPS combined with GRACE, 0.701 (95% CI 0.569-0.813) for the GPS combined with the PLRS, 0.705 (95% CI 0.571-0.816) for GRACE combined with the PLRS, and 0.761 (95% CI 0.634-0.862) for the GPS combined with GRACE and the PLRS. Compared with the RGPS alone, the combined diagnostic model of the GPS plus GRACE and the PLRS and the combined diagnostic model of the GPS plus GRACE had a higher AUC value (P = 0.0184 and P = 0.0156, respectively). In the nonacute infection group, the AUCs were 0.731 (95% CI 0.640-0.809) for the GPS, 0.726 (95% CI 0.635-0.805) for the RGPS, 0.510 (95% CI 0.415-0.604) for the PLRS, and 0.786 (95% CI 0.700-0.857) for GRACE. This AUC was 0.802 (95% CI 0.717-0.870) for the GPS combined with GRACE, 0.760 (95% CI 0.672-0.835) for the GPS combined with the PLRS, 0.811 (95% CI 0.728-0.878) for GRACE combined with the PLRS, and 0.832 (95% CI 0.751-0.895) for the GPS combined with GRACE and the PLRS. The combined diagnostic model of the GPS plus GRACE and the PLRS seemed to have a higher AUC value than each single score, but there was not statistically significantly different.

## DISCUSSION

This present study investigated the predictive ability of the GPS, PLRS and GRACE for the MACEs during hospitalization in patients with acute myocardial infarction. The main findings are listed below. (1) The GPS and GRACE had good predictive ability for the MACEs during hospitalization, multivariate logistic regression analysis showed that they were both independent risk factors for MACEs, and the combined diagnostic model of the GPS and GRACE tended to have a higher predictive ability than each individual score. (2) The PLRS could not effectively predict the occurrence of MACEs during hospitalization, but the diagnostic model of the PLRS combined with the GPS and GRACE had a better predictive ability than each individual score.

Although GRACE is considered to have some predictive power for the risk of hospitalization and 5-year mortality in ACS patients [6], its composition lacks an inflammation assessment component. Two large-scale randomized controlled trials, COLCOT [23] and CANTOS [24], were designed to investigate the efficacy of anti-inflammatory therapy in patients who had myocardial infarction with high H-CRP levels, and they were found to reduce the incidence of MACEs. Another recent large-scale clinical randomized controlled trial, LoDoCo2 [25], was designed to investigate the efficacy of colchicine for long-term outcomes in patients with chronic coronary disease, the results of which showed that long-term anti-inflammatory therapy could reduce the occurrence of cardiovascular events, and that the patients started to show benefit manifestations at the beginning of the intervention, and the benefit increased gradually and further with the duration of treatment. Our previous study^7^ also showed that the GPS has similar predictive power to GRACE in predicting MACEs in patients with AMI. In addition to demonstrating, once again, that the GPS had a predictive ability that is not inferior to that of GRACE and that the GPS was an independent risk factor for the MACEs, the results of this study were also the first to explore the predictive ability of the combined diagnostic model of the GPS plus GRACE through ROC analysis; the combined model tended to have a higher predictive value than each individual score, although the differences were not statistically significant. In addition, the cutoff values of H-CRP and albumin in the GPS were derived using prognosis data from cancer patients. Therefore, another aim of this present study was to investigate the cutoff values of H-CRP and albumin suitable for the prognosis of patients with AMI. Unfortunately, RGPS, as defined by the data from previous studies, was slightly less predictive of MACEs in AMI patients than the GPS in both the overall and subgroup results. The possible reason for this is that our previous study also had a small sample size, so the cutoff values for H-CRP and albumin in the RGPS were not accurately obtained. Although further optimization of the GPS has not been completed, this finding also implies that this current GPS has a good predictive value for the prognosis of patients with AMI.

Thrombosis is also considered a key factor affecting the prognosis of patients with MI. Platelet count is associated with the risk of cardiovascular events, and its elevation is thought to be associated with thrombosis [7, 26, 27]. Lymphocyte counts, in addition to exerting immune functions, also been suggested to be associated with prognosis in patients with coronary artery disease (CAD). Drobni et al. [28] investigated lymphocyte counts in relation to patients with myocarditis and found that low levels of lymphocyte counts were associated with the occurrence of MACEs. Another prospective cohort study [29] aimed to investigate the ability of leukocytes to predict long-term outcome in patients with multivessel CAD, and its results also suggested that lymphocyte count was an independent risk factor for death. PLR, as the ratio of platelet count to lymphocyte count, has recently been reported by several studies to have a good ability to predict the prognosis of patients with ACS [12–14]. Arcy et al. [14] found that a high PLR (>137) was associated with a higher in-hospital risk MACEs than a low PLR (<137) in patients with AMI, and the PLR was an independent risk factor for MACEs. Additionally, Maimaiti et al. [12] found that a high PLR (>165.33) was associated with a higher in-hospital risk of MACEs than a low PLR (<165.33) in patients with MI with anterior descending artery disease. However, our present study found that the PLRS could not effectively predict MACEs in patients with AMI. Part of the reason may be that the definition of MACEs as a composite endpoint differed in different studies, and the trials by Arcy et al. and/or Maimaiti et al. did not include outcomes such as stroke and cardiovascular death. The different populations included may also be one of the reasons. Maimaiti et al. included only patients with anterior descending artery disease and myocardial infarction. In other words, patients with single vessel disease were included. Compared with patients with multiple vessel disease, the difference in coronary microcirculation function and ischemic preconditioning may have an impact on the outcome. In addition, the PLRS in this study was derived from the data from our previous study because it belonged to a small sample trial, so the selection of cutoff values for platelets and lymphocytes in the PLRS were not accurate, and MACEs could not be predicted statistically by redefining the cutoff values of the PLRS (Arcy) to 137 or (Maimaiti) 165.33 (Supplementary Figure 5 and Supplementary Table 8). Another important reason may be the different follow-up times in the various studies. A prospective cohort study [13] showed that with the increase of follow-up time, the incidence of cardiovascular events in patients with a high PLR was higher than that in patients with a low PLR. The results of another study [30] also showed that during hospitalization, the level of PLR had no correlation with cardiovascular mortality, whereas after one month of follow-up, high levels of PLR seemed to have higher cardiovascular mortality than low levels of PLR. And after six months of follow-up, high-level PLR had higher cardiovascular mortality compared with low-level PLR accompanied by statistical difference. Interestingly, the diagnostic model of GPS combined with GRACE tended to have higher predictive value than individual scores. But building into a triple diagnostic model consisting of GPS, GRACE and PLRS could further improve prediction of risks of MACEs. Although some studies have indicated the PLRS to be a possible indicator of the long-term prognosis of cardiovascular events, there is controversy about the predictive ability of MACEs during hospitalization [12, 14, 30]. Our results showed that although the PLRS could not independently predict MACEs during hospitalization, it could further optimize the predictive ability of the combined model with the GPS and GRACE. We also revisited data from the previous study^7^ to assess the predictive value of GPS, GRACE and PLRS for AMI and found similar results (data not shown).

The results of the subgroup analysis suggested that both the GPS and GRACE had similar predictive abilities for MACEs during hospitalization, regardless of inclusion in the STEMI and NSTEMI group, the PCI or non-PCI group, or the infection or noninfection group, while the PLRS could not effectively predict MACEs. Compared with the RGPS, the GPS still seemed to have better prediction ability. In addition, pairwise comparisons in the multivariate ROC analysis were performed for each subgroup, and the results were generally consistent with the overall results, which demonstrated the robustness of the results of this study.

The current study has some limitations. First, it was a single-center, small-sample trial. Second, we only observed MACEs during hospitalization, and long-term follow-up would give a more comprehensive evaluation. In addition, the optimization of the cutoff values for H-CRP and albumin in the GPS was not completed, and the cutoff values of the PLRS varied greatly among different studies, which may have affected the prediction of MACEs by the joint diagnostic model.

Overall, our study showed that the combined diagnostic model including the GPS plus GRACE and the PLRS had better predictive ability for MACEs during hospitalization compared with that of each individual score. Thus, the use of a combined model with the GPS plus GRACE and the PLRS will be of clinical benefit in a broad group of individuals with AMI. However, large, multicenter, and prospective studies still need to be performed to clarify the predictive power of the combined diagnostic model for patients with AMI during hospitalization and follow-up and to further optimize this model.

## Supplementary Material

Supplementary Materials
