# Supplementary material for: Predictive value of inflammation-based Glasgow prognostic score, platelet-lymphocyte ratio, and global registry of acute coronary events score for major cardiovascular and cerebrovascular events during hospitalization in patients with acute myocardial infarction
Source: Aging (Albany NY). 2021 Jul 16;13(14):18274–86. doi: 10.18632/aging.203273 (PMC8351676; doi:10.18632/aging.203273)
Supplement: Supplementary Materials [file aging-13-203273-s001.pdf]

Supplementary Materials

Predictive value of inflammation-based Glasgow Prognostic Score, platelet-lymphocyte ratio, and Global Registry of Acute Coronary Events score for major cardiovascular and cerebrovascular events during hospitalization in patients with acute myocardial infarction.

Supplementary Figure 1. Design of the present study.. 3

Supplementary Table 1. Statistics of missing value and extremesa 4

Supplementary Table 2. Pairwise comparison of univariate receiver operating characteristic curves 6

Supplementary Table 3. Pairwise comparison of multivariate receiver operating characteristic curves 8

Supplementary Table 4. Comparisons between multivariate receiver operating characteristic (ROC) curves with high scores and univariate ROC curves 10

Supplementary Figure 2. Subgroup results based on type of myocardial infarction (MI) in patients 12

Supplementary Table 5. Subgroup results based on type of myocardial infarction (MI) in patients 14

Supplementary Figure 3. Subgroup results based on whether the patients undergoing percutaneous coronary intervention (PCI) or not 22

Supplementary Table 6. Subgroup results based on whether the patients undergoing percutaneous coronary intervention (PCI) or not 24

Supplementary Figure 4. Subgroup results based on whether the patients had an acute infection or not 31

Supplementary Table 7. Subgroup results based on whether the patients had an acute infection or not 33

Supplementary Figure 5. Receiver operating characteristic of three PLRSs 41

Supplementary Table 8. Receiver operating characteristic of three PLRSs 42

Supplementary Figure 1. Design of the present study.

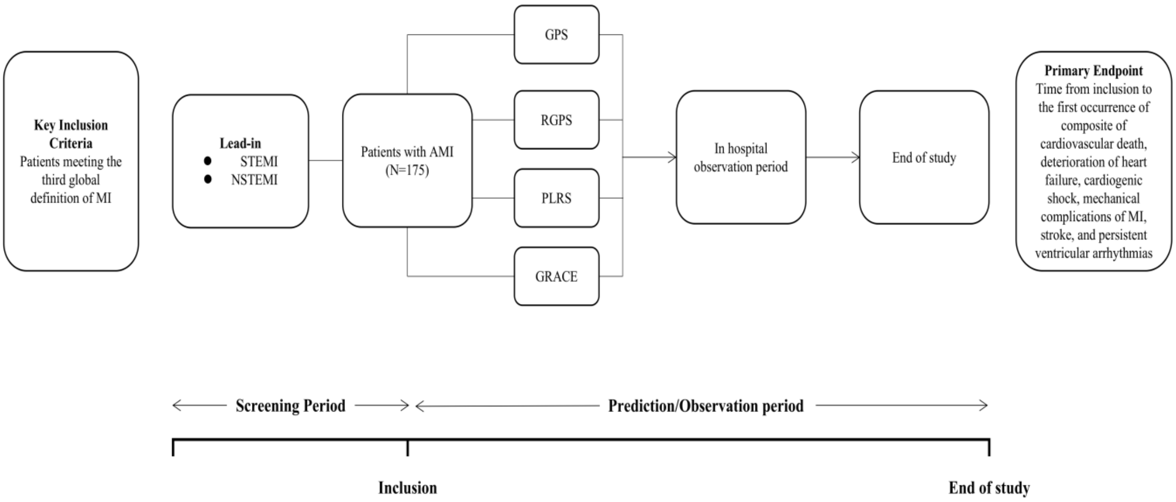

AMI = Acute MI; MI = myocardial infarction; NSTEMI = non-ST-elevation myocardial infarction; GPS = inflammation-based Glasgow Prognostic Score; GRACE = Global Registry of Acute Coronary Events; PLRS = platelet-to-lymphocyte ratio score; RGPS = redefined GPS; STEMI = ST-elevation myocardial infarction.

Supplementary Table 1. Statistics of missing value and extremesa.

	N	Mean	Std. Deviation	Missing		No. of Extremesa	
				Count	Percent	Low	High
Age	175	65.72	15.900	0	0.0	0	0
Lymphocyte	175	1.3535	0.59552	0	0.0	0	4
Hemoglobin	175	125.75	19.965	0	0.0	2	0
Platelet	175	198.79	72.370	0	0.0	1	10
ALT	175	44.22	45.215	0	0.0	0	10
CK	175	1060.17	1415.633	0	0.0	0	19
CK-MB	175	90.27	123.282	0	0.0	0	16
Albumin	175	36.249	4.5713	0	0.0	4	1
LDL	172	2.3278	0.87149	3	1.7	0	6
Creatinine	175	105.665	83.6032	0	0.0	0	12
HS-CRP	175	25.9226	38.82788	0	0.0	0	26
BNP	169	571.48	762.106	6	3.4	0	8
TNI	175	66.5725	171.14042	0	0.0	0	24
Heart rate	175	81.64	16.708	0	0.0	0	2
SBP	175	131.83	22.842	0	0.0	1	1
DBP	175	74.81	13.883	0	0.0	0	3
D-Dimer	172	1.37998	2.935950	3	1.7	0	17
Males	175			0	0.0		
ST segment down	175			0	0.0		
MACE	175			0	0.0		
Hypertension	175			0	0.0		
Diabetics	175			0	0.0		

Killip class	175			0	0.0		
PCI type	175			0	0.0		
Acute infections	175			0	0.0		
Autoimmune diseases	175			0	0.0		
Nephrotic syndrome or uremia	175			0	0.0		
Liver cirrhosis	175			0	0.0		

a Number of cases outside the range (Q1 - 1.5*IQR, Q3 + 1.5*IQR).

Supplementary Table 2. Pairwise comparison of univariate receiver operating characteristic curves.

GPS ~ RGPS	
Difference between areas	0.0144
Standard Error [a]	0.0111
95% Confidence Interval	-0.00738 to 0.0361
z statistic	1.294
Significance level	P = 0.1956
GPS ~ GRACE	
Difference between areas	0.0133
Standard Error [a]	0.0427
95% Confidence Interval	-0.0704 to 0.0971
z statistic	0.312
Significance level	P = 0.7550
GPS ~ PLRS	
Difference between areas	0.219
Standard Error [a]	0.0543
95% Confidence Interval	0.112 to 0.325
z statistic	4.026
Significance level	P = 0.0001
RGPS ~ GRACE	
Difference between areas	0.0277
Standard Error [a]	0.0420
95% Confidence Interval	-0.0547 to 0.110
z statistic	0.659
Significance level	P = 0.5099
RGPS ~ PLRS	
Difference between areas	0.204
Standard Error [a]	0.0538
95% Confidence Interval	0.0991 to 0.310
z statistic	3.803
Significance level	P = 0.0001
GRACE ~ PLRS	

Difference between areas	0.232
Standard Error [a]	0.0551
95% Confidence Interval	0.124 to 0.340
z statistic	4.213
Significance level	P < 0.0001

Supplementary Table 3. Pairwise comparison of multivariate receiver operating characteristic curves.

GPS_GRACE ~ GPS_PLRS	
Difference between areas	0.0266
Standard Error [a]	0.0243
95% Confidence Interval	-0.0210 to 0.0742
z statistic	1.094
Significance level	P = 0.2739
GPS_GRACE ~ GRACE_PLRS	
Difference between areas	0.0157
Standard Error [a]	0.0330
95% Confidence Interval	-0.0489 to 0.0804
z statistic	0.477
Significance level	P = 0.6333
GPS_GRACE ~ GPS_GRACE_PLRS	
Difference between areas	0.0317
Standard Error [a]	0.0139
95% Confidence Interval	0.00445 to 0.0589
z statistic	2.281
Significance level	P = 0.0226
GPS_PLRS ~ GRACE_PLRS	
Difference between areas	0.0108
Standard Error [a]	0.0481
95% Confidence Interval	-0.0835 to 0.105
z statistic	0.225
Significance level	P = 0.8219
GPS_PLRS ~ GPS_GRACE_PLRS	
Difference between areas	0.0582
Standard Error [a]	0.0311
95% Confidence Interval	-0.00278 to 0.119
z statistic	1.871
Significance level	P = 0.0614
GRACE_PLRS ~ GPS_GRACE_PLRS	

Difference between areas	0.0474
Standard Error [a]	0.0232
95% Confidence Interval	0.00199 to 0.0928
z statistic	2.046
Significance level	P = 0.0408

Supplementary Table 4. Comparisons between multivariate receiver operating characteristic (ROC) curves with high scores and univariate ROC curves.

GPS_GRACE_PLRS ~ GPS	
Difference between areas	0.0611
Standard Error [a]	0.0249
95% Confidence Interval	0.0123 to 0.110
z statistic	2.456
Significance level	P = 0.0140
GPS_GRACE_PLRS ~ RGPS	
Difference between areas	0.0755
Standard Error [a]	0.0260
95% Confidence Interval	0.0246 to 0.126
z statistic	2.908
Significance level	P = 0.0036
GPS_GRACE_PLRS ~ GRACE	
Difference between areas	0.0478
Standard Error [a]	0.0230
95% Confidence Interval	0.00271 to 0.0928
z statistic	2.078
Significance level	P = 0.0377
GPS_GRACE ~ GPS	
Difference between areas	0.0294
Standard Error [a]	0.0158
95% Confidence Interval	-0.00161 to 0.0605
z statistic	1.858
Significance level	P = 0.0631
GPS_GRACE ~ RGPS	
Difference between areas	0.0438
Standard Error [a]	0.0183
95% Confidence Interval	0.00800 to 0.0796
z statistic	2.398
Significance level	P = 0.0165
GPS_GRACE ~ GRACE	

Difference between areas	0.0161
Standard Error [a]	0.0328
95% Confidence Interval	-0.0481 to 0.0803
z statistic	0.492
Significance level	P = 0.6230

Supplementary Figure 2. Subgroup results based on type of myocardial infarction (MI) in patients.

(1) Univariate receiver operating characteristic (ROC) curve analysis in STEMI group.

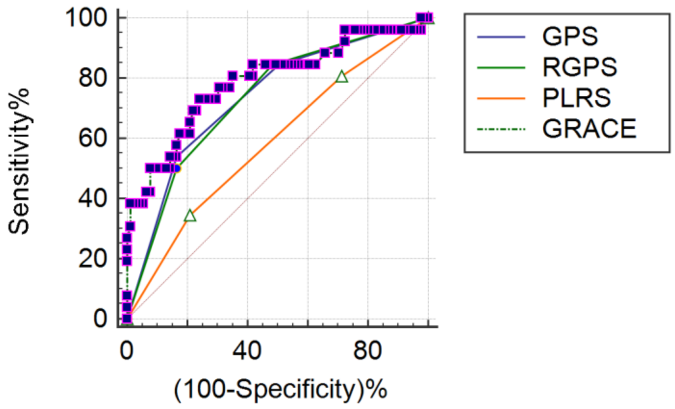

(2) Multivariate ROC curve analysis in STEMI group.

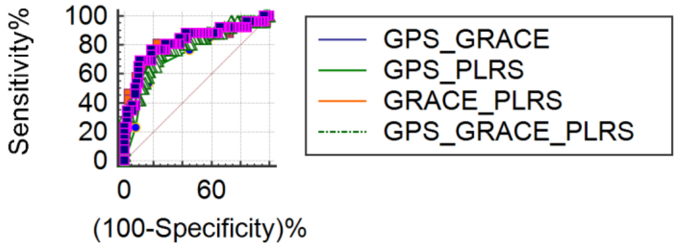

(3) Univariate ROC curve analysis in NSTEMI group.

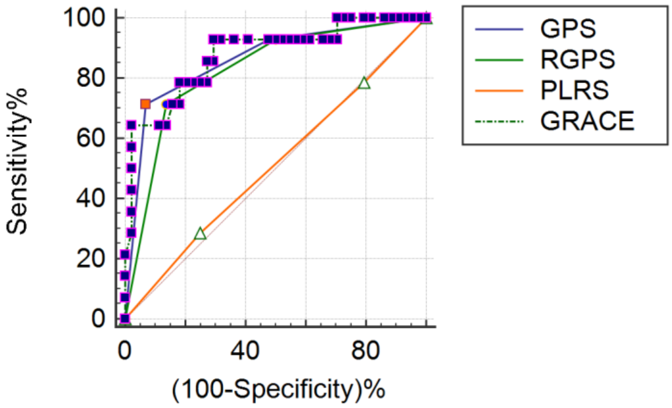

(4) Multivariate ROC curve analysis in NSTEMI group.

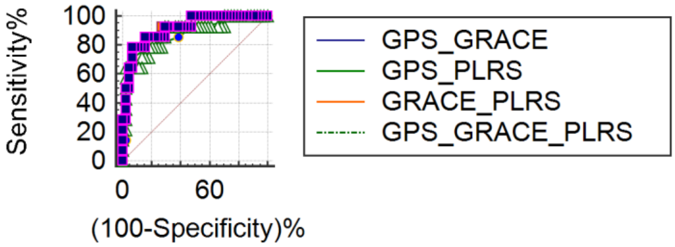

Supplementary Table 5. Subgroup results based on type of myocardial infarction (MI) in patients.

(1) Pairwise comparison of univariate receiver operating characteristic (ROC) curves in STEMI group.

Variable	AUC	SE [a]	95% CI [b]
GPS	0.737	0.0533	0.647 to 0.814
RGPS	0.732	0.0525	0.643 to 0.810
PLRS	0.586	0.0600	0.491 to 0.676
GRACE	0.788	0.0568	0.703 to 0.858

GPS ~ RGPS	
Difference between areas	0.00423
Standard Error [a]	0.0144
95% Confidence Interval	-0.0239 to 0.0324
z statistic	0.294
Significance level	P = 0.7686
**GPS ~ PLRS**	
Difference between areas	0.151
Standard Error [a]	0.0635
95% Confidence Interval	0.0262 to 0.275
z statistic	2.373
Significance level	P = 0.0177
**GPS ~ GRACE**	
Difference between areas	0.0516
Standard Error [a]	0.0488
95% Confidence Interval	-0.0441 to 0.147
z statistic	1.057
Significance level	P = 0.2907
**RGPS ~ PLRS**	
Difference between areas	0.146
Standard Error [a]	0.0628
95% Confidence Interval	0.0234 to 0.269
z statistic	2.334

Significance level	P = 0.0196
RGPS ~ GRACE	
Difference between areas	0.0558
Standard Error [a]	0.0484
95% Confidence Interval	-0.0391 to 0.151
z statistic	1.153
Significance level	P = 0.2490
PLRS ~ GRACE	
Difference between areas	0.202
Standard Error [a]	0.0640
95% Confidence Interval	0.0767 to 0.328
z statistic	3.158
Significance level	P = 0.0016

(2) Pairwise comparison of multivariate ROC curves in STEMI group.

Variable	AUC	SE [a]	95% CI [b]
GPS_GRACE	0.807	0.0569	0.724 to 0.874
GPS_PLRS	0.738	0.0586	0.649 to 0.815
GRACE_PLRS	0.791	0.0556	0.706 to 0.860
GPS_GRACE_PLRS	0.816	0.0550	0.734 to 0.882

GPS_GRACE ~ GPS_PLRS	
Difference between areas	0.0693
Standard Error [a]	0.0362
95% Confidence Interval	-0.00165 to 0.140
z statistic	1.914
Significance level	P = 0.0556
GPS_GRACE ~ GRACE_PLRS	
Difference between areas	0.0167
Standard Error [a]	0.0206
95% Confidence Interval	-0.0238 to 0.0572
z statistic	0.808
Significance level	P = 0.4188

GPS_GRACE ~ GPS_GRACE_PLRS	
Difference between areas	0.00866
Standard Error [a]	0.00870
95% Confidence Interval	-0.00838 to 0.0257
z statistic	0.996
Significance level	P = 0.3191
**GPS_PLRS ~ GRACE_PLRS**	
Difference between areas	0.0526
Standard Error [a]	0.0520
95% Confidence Interval	-0.0494 to 0.155
z statistic	1.011
Significance level	P = 0.3119
**GPS_PLRS ~ GPS_GRACE_PLRS**	
Difference between areas	0.0780
Standard Error [a]	0.0398
95% Confidence Interval	-0.0000320 to 0.156
z statistic	1.959
Significance level	P = 0.0501
**GRACE_PLRS ~ GPS_GRACE_PLRS**	
Difference between areas	0.0254
Standard Error [a]	0.0202
95% Confidence Interval	-0.0143 to 0.0650
z statistic	1.254
Significance level	P = 0.2097

(3) Comparisons between multivariate ROC curves with high scores and univariate ROC curves in STEMI group.

Variable	AUC	SE [a]	95% CI [b]
GPS_GRACE_PLRS	0.816	0.0550	0.734 to 0.882
GPS	0.737	0.0533	0.647 to 0.814
RGPS	0.732	0.0525	0.643 to 0.810
GRACE	0.788	0.0568	0.703 to 0.858

GPS_GRACE_PLRS ~ GPS

Difference between areas	0.0792
Standard Error [a]	0.0359
95% Confidence Interval	0.00883 to 0.150
z statistic	2.206
Significance level	P = 0.0274
GPS_GRACE_PLRS ~ RGPS	
Difference between areas	0.0835
Standard Error [a]	0.0368
95% Confidence Interval	0.0114 to 0.156
z statistic	2.271
Significance level	P = 0.0231
GPS_GRACE_PLRS ~ GRACE	
Difference between areas	0.0277
Standard Error [a]	0.0195
95% Confidence Interval	-0.0106 to 0.0660
z statistic	1.418
Significance level	P = 0.1562

(4) Pairwise comparison of univariate receiver ROC curves in NSTEMI group.

Variable	AUC	SE [a]	95% CI [b]
GPS	0.864	0.0585	0.749 to 0.940
RGPS	0.830	0.0608	0.708 to 0.916
PLRS	0.511	0.0848	0.376 to 0.644
GRACE	0.877	0.0574	0.764 to 0.948

GPS ~ RGPS	
Difference between areas	0.0349
Standard Error [a]	0.0187
95% Confidence Interval	-0.00180 to 0.0716
z statistic	1.864
Significance level	P = 0.0624
GPS ~ PLRS	
Difference between areas	0.354

Standard Error [a]	0.100
95% Confidence Interval	0.158 to 0.550
z statistic	3.533
Significance level	P = 0.0004
GPS ~ GRACE	
Difference between areas	0.0122
Standard Error [a]	0.0728
95% Confidence Interval	-0.131 to 0.155
z statistic	0.167
Significance level	P = 0.8672
RGPS ~ PLRS	
Difference between areas	0.319
Standard Error [a]	0.0995
95% Confidence Interval	0.124 to 0.514
z statistic	3.205
Significance level	P = 0.0014
RGPS ~ GRACE	
Difference between areas	0.0471
Standard Error [a]	0.0735
95% Confidence Interval	-0.0970 to 0.191
z statistic	0.640
Significance level	P = 0.5219
PLRS ~ GRACE	
Difference between areas	0.366
Standard Error [a]	0.0965
95% Confidence Interval	0.177 to 0.555
z statistic	3.792
Significance level	P = 0.0001

(5) Pairwise comparison of multivariate ROC curves in NSTEMI group.

Variable	AUC	SE [a]	95% CI [b]
GPS_GRACE	0.913	0.0424	0.809 to 0.971
GPS_PLRS	0.869	0.0607	0.755 to 0.943
GRACE_PLRS	0.877	0.0574	0.764 to 0.948

GPS_GRACE_PLRS	0.912	0.0428	0.808 to 0.971

GPS_GRACE ~ GPS_PLRS	
Difference between areas	0.0438
Standard Error [a]	0.0509
95% Confidence Interval	-0.0560 to 0.144
z statistic	0.861
Significance level	P = 0.3894
GPS_GRACE ~ GRACE_PLRS	
Difference between areas	0.0365
Standard Error [a]	0.0355
95% Confidence Interval	-0.0330 to 0.106
z statistic	1.030
Significance level	P = 0.3028
GPS_GRACE ~ GPS_GRACE_PLRS	
Difference between areas	0.000812
Standard Error [a]	0.00262
95% Confidence Interval	-0.00432 to 0.00594
z statistic	0.310
Significance level	P = 0.7565
GPS_PLRS ~ GRACE_PLRS	
Difference between areas	0.00731
Standard Error [a]	0.0792
95% Confidence Interval	-0.148 to 0.162
z statistic	0.0923
Significance level	P = 0.9265
GPS_PLRS ~ GPS_GRACE_PLRS	
Difference between areas	0.0430
Standard Error [a]	0.0497
95% Confidence Interval	-0.0544 to 0.140
z statistic	0.865
Significance level	P = 0.3869
GRACE_PLRS ~ GPS_GRACE_PLRS	
Difference between areas	0.0357

Standard Error [a]	0.0365
95% Confidence Interval	-0.0359 to 0.107
z statistic	0.978
Significance level	P = 0.3283

(6) Comparisons between multivariate ROC curves with high scores and univariate ROC curves in NSTEMI group.

Variable	AUC	SE [a]	95% CI [b]
GPS_GRACE_PLRS	0.912	0.0428	0.808 to 0.971
GPS_GRACE	0.913	0.0424	0.809 to 0.971
GPS	0.864	0.0585	0.749 to 0.940
RGPS	0.830	0.0608	0.708 to 0.916
GRACE	0.877	0.0574	0.764 to 0.948

GPS_GRACE_PLRS ~ GPS	
Difference between areas	0.0479
Standard Error [a]	0.0400
95% Confidence Interval	-0.0304 to 0.126
z statistic	1.198
Significance level	P = 0.2308
GPS_GRACE_PLRS ~ RGPS	
Difference between areas	0.0828
Standard Error [a]	0.0419
95% Confidence Interval	0.000611 to 0.165
z statistic	1.975
Significance level	P = 0.0483
GPS_GRACE_PLRS ~ GRACE	
Difference between areas	0.0357
Standard Error [a]	0.0367
95% Confidence Interval	-0.0362 to 0.108
z statistic	0.973
Significance level	P = 0.3303

GPS_GRACE ~ GPS	
Difference between areas	0.0487
Standard Error [a]	0.0413
95% Confidence Interval	-0.0323 to 0.130
z statistic	1.178
Significance level	P = 0.2386
**GPS_GRACE ~ RGPS**	
Difference between areas	0.0836
Standard Error [a]	0.0431
95% Confidence Interval	-0.000968 to 0.168
z statistic	1.938
Significance level	P = 0.0527
**GPS_GRACE ~ GRACE**	
Difference between areas	0.0365
Standard Error [a]	0.0356
95% Confidence Interval	-0.0333 to 0.106
z statistic	1.025
Significance level	P = 0.3055

Supplementary Figure 3. Subgroup results based on whether the patients undergoing percutaneous coronary intervention (PCI) or not.

(1) Univariate receiver operating characteristic (ROC) curve analysis in PCI group.

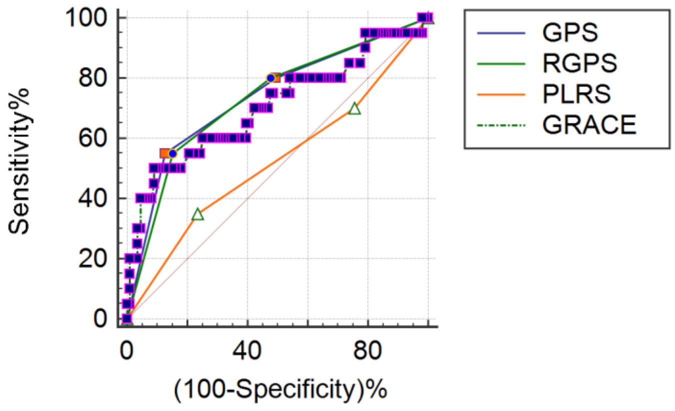

(2) Multivariate ROC curve analysis in PCI group.

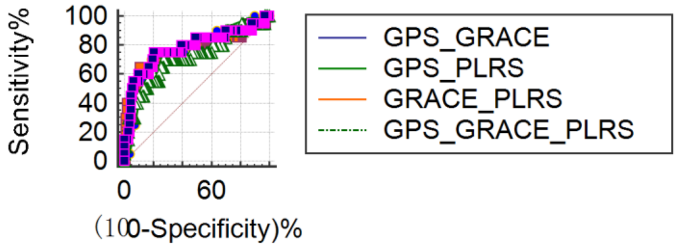

(3) Univariate ROC curve analysis in non-PCI group.

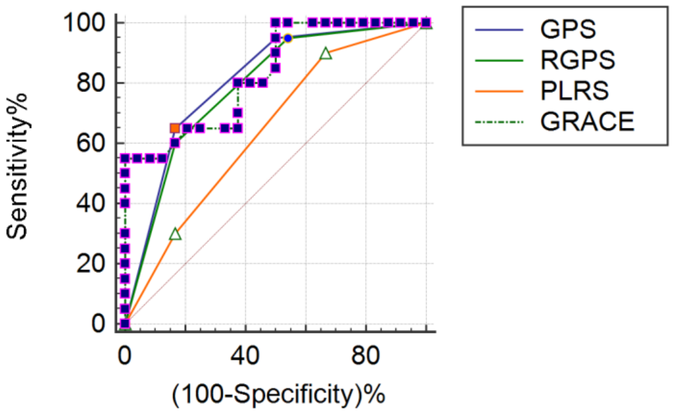

(4) Multivariate ROC curve analysis in non-PCI group.

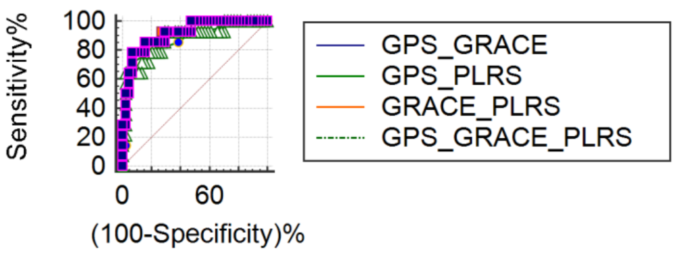

Supplementary Table 6. Subgroup results based on whether the patients undergoing percutaneous coronary intervention (PCI) or not.

(1) Pairwise comparison of univariate receiver operating characteristic (ROC) curves in PCI group.

Variable	AUC	SE [a]	95% CI [b]
GPS	0.738	0.0636	0.654 to 0.811
RGPS	0.731	0.0621	0.647 to 0.805
PLRS	0.522	0.0737	0.433 to 0.610
GRACE	0.704	0.0741	0.618 to 0.781

GPS ~ RGPS	
Difference between areas	0.00676
Standard Error [a]	0.0109
95% Confidence Interval	-0.0146 to 0.0281
z statistic	0.620
Significance level	P = 0.5354
**GPS ~ PLRS**	
Difference between areas	0.216
Standard Error [a]	0.0723
95% Confidence Interval	0.0742 to 0.358
z statistic	2.986
Significance level	P = 0.0028
**GPS ~ GRACE**	
Difference between areas	0.0338
Standard Error [a]	0.0561
95% Confidence Interval	-0.0762 to 0.144
z statistic	0.602
Significance level	P = 0.5472
**RGPS ~ PLRS**	
Difference between areas	0.209
Standard Error [a]	0.0716
95% Confidence Interval	0.0689 to 0.350
z statistic	2.923

Significance level	P = 0.0035
RGPS ~ GRACE	
Difference between areas	0.0270
Standard Error [a]	0.0558
95% Confidence Interval	-0.0823 to 0.136
z statistic	0.485
Significance level	P = 0.6279
PLRS ~ GRACE	
Difference between areas	0.182
Standard Error [a]	0.0823
95% Confidence Interval	0.0208 to 0.344
z statistic	2.213
Significance level	P = 0.0269

(2) Pairwise comparison of multivariate ROC curves in PCI group.

Variable	AUC	SE [a]	95% CI [b]
GPS_GRACE	0.760	0.0747	0.677 to 0.830
GPS_PLRS	0.763	0.0597	0.681 to 0.833
GRACE_PLRS	0.718	0.0721	0.633 to 0.793
GPS_GRACE_PLRS	0.776	0.0699	0.695 to 0.844

GPS_GRACE ~ GPS_PLRS	
Difference between areas	0.00360
Standard Error [a]	0.0383
95% Confidence Interval	-0.0715 to 0.0787
z statistic	0.0941
Significance level	P = 0.9251
GPS_GRACE ~ GRACE_PLRS	
Difference between areas	0.0417
Standard Error [a]	0.0482
95% Confidence Interval	-0.0529 to 0.136
z statistic	0.864
Significance level	P = 0.3878

GPS_GRACE ~ GPS_GRACE_PLRS	
Difference between areas	0.0167
Standard Error [a]	0.0158
95% Confidence Interval	-0.0143 to 0.0476
z statistic	1.055
Significance level	P = 0.2916
GPS_PLRS ~ GRACE_PLRS	
Difference between areas	0.0453
Standard Error [a]	0.0627
95% Confidence Interval	-0.0777 to 0.168
z statistic	0.721
Significance level	P = 0.4706
GPS_PLRS ~ GPS_GRACE_PLRS	
Difference between areas	0.0131
Standard Error [a]	0.0316
95% Confidence Interval	-0.0488 to 0.0749
z statistic	0.414
Significance level	P = 0.6789
GRACE_PLRS ~ GPS_GRACE_PLRS	
Difference between areas	0.0583
Standard Error [a]	0.0420
95% Confidence Interval	-0.0240 to 0.141
z statistic	1.388
Significance level	P = 0.1651

(3) Comparisons between multivariate ROC curves with high scores and univariate ROC curves in PCI groupPairwise comparison of univariate receiver operating characteristic (ROC) curves in PCI group.

Variable	AUC	SE [a]	95% CI [b]
GPS_GRACE_PLRS	0.776	0.0699	0.695 to 0.844
GPS	0.738	0.0636	0.654 to 0.811
RGPS	0.731	0.0621	0.647 to 0.805
GRACE	0.704	0.0741	0.618 to 0.781

GPS_GRACE_PLRS ~ GPS

Difference between areas	0.0383
Standard Error [a]	0.0299
95% Confidence Interval	-0.0204 to 0.0969
z statistic	1.279
Significance level	P = 0.2007
GPS_GRACE_PLRS ~ RGPS	
Difference between areas	0.0450
Standard Error [a]	0.0313
95% Confidence Interval	-0.0163 to 0.106
z statistic	1.440
Significance level	P = 0.1499
GPS_GRACE_PLRS ~ GRACE	
Difference between areas	0.0721
Standard Error [a]	0.0382
95% Confidence Interval	-0.00281 to 0.147
z statistic	1.887
Significance level	P = 0.0592

(4) Pairwise comparison of univariate ROC curves in non-PCI group.

Variable	AUC	SE [a]	95% CI [b]
GPS	0.808	0.0619	0.662 to 0.911
RGPS	0.788	0.0644	0.638 to 0.896
PLRS	0.642	0.0752	0.483 to 0.780
GRACE	0.828	0.0614	0.684 to 0.925

GPS ~ RGPS	
Difference between areas	0.0208
Standard Error [a]	0.0157
95% Confidence Interval	-0.00987 to 0.0515
z statistic	1.330
Significance level	P = 0.1835
GPS ~ PLRS	
Difference between areas	0.167

Standard Error [a]	0.0886
95% Confidence Interval	-0.00699 to 0.340
z statistic	1.881
Significance level	P = 0.0600
GPS ~ GRACE	
Difference between areas	0.0198
Standard Error [a]	0.0781
95% Confidence Interval	-0.133 to 0.173
z statistic	0.253
Significance level	P = 0.8001
RGPS ~ PLRS	
Difference between areas	0.146
Standard Error [a]	0.0898
95% Confidence Interval	-0.0302 to 0.322
z statistic	1.624
Significance level	P = 0.1044
RGPS ~ GRACE	
Difference between areas	0.0406
Standard Error [a]	0.0753
95% Confidence Interval	-0.107 to 0.188
z statistic	0.540
Significance level	P = 0.5894
PLRS ~ GRACE	
Difference between areas	0.186
Standard Error [a]	0.0961
95% Confidence Interval	-0.00187 to 0.375
z statistic	1.940
Significance level	P = 0.0523

(5) Pairwise comparison of multivariate ROC curves in non-PCI group.

Variable	AUC	SE [a]	95% CI [b]
GPS_GRACE	0.856	0.0560	0.718 to 0.944
GPS_PLRS	0.826	0.0687	0.682 to 0.924
GRACE_PLRS	0.834	0.0603	0.692 to 0.929

GPS_GRACE_PLRS	0.862	0.0576	0.725 to 0.948

GPS_GRACE ~ GPS_PLRS	
Difference between areas	0.0302
Standard Error [a]	0.0411
95% Confidence Interval	-0.0504 to 0.111
z statistic	0.735
Significance level	P = 0.4625
**GPS_GRACE ~ GRACE_PLRS**	
Difference between areas	0.0219
Standard Error [a]	0.0444
95% Confidence Interval	-0.0651 to 0.109
z statistic	0.493
Significance level	P = 0.6221
**GPS_GRACE ~ GPS_GRACE_PLRS**	
Difference between areas	0.00625
Standard Error [a]	0.0213
95% Confidence Interval	-0.0356 to 0.0481
z statistic	0.293
Significance level	P = 0.7696
**GPS_PLRS ~ GRACE_PLRS**	
Difference between areas	0.00833
Standard Error [a]	0.0591
95% Confidence Interval	-0.108 to 0.124
z statistic	0.141
Significance level	P = 0.8879
**GPS_PLRS ~ GPS_GRACE_PLRS**	
Difference between areas	0.0365
Standard Error [a]	0.0293
95% Confidence Interval	-0.0211 to 0.0940
z statistic	1.243
Significance level	P = 0.2140
**GRACE_PLRS ~ GPS_GRACE_PLRS**	
Difference between areas	0.0281

Standard Error [a]	0.0376
95% Confidence Interval	-0.0456 to 0.102
z statistic	0.747
Significance level	P = 0.4548

(6) Comparisons between multivariate ROC curves with high scores and univariate ROC curves in non-PCI group.

Variable	AUC	SE [a]	95% CI [b]
GPS_GRACE_PLRS	0.862	0.0576	0.725 to 0.948
GPS	0.808	0.0619	0.662 to 0.911
RGPS	0.788	0.0644	0.638 to 0.896
GRACE	0.828	0.0614	0.684 to 0.925

GPS_GRACE_PLRS ~ GPS	
Difference between areas	0.0542
Standard Error [a]	0.0362
95% Confidence Interval	-0.0167 to 0.125
z statistic	1.498
Significance level	P = 0.1342
**GPS_GRACE_PLRS ~ RGPS**	
Difference between areas	0.0750
Standard Error [a]	0.0370
95% Confidence Interval	0.00239 to 0.148
z statistic	2.024
Significance level	P = 0.0429
**GPS_GRACE_PLRS ~ GRACE**	
Difference between areas	0.0344
Standard Error [a]	0.0589
95% Confidence Interval	-0.0810 to 0.150
z statistic	0.584
Significance level	P = 0.5594

Supplementary Figure 4. Subgroup results based on whether the patients had an acute infection or not.

(1) Univariate receiver operating characteristic (ROC) curve analysis in acute infection group.

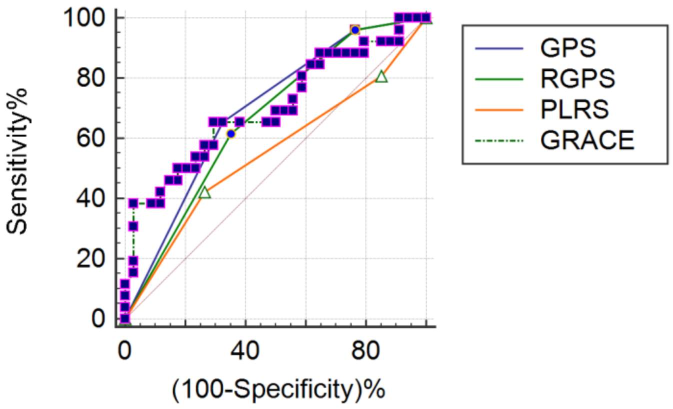

(2) Multivariate ROC curve analysis in acute infection group.

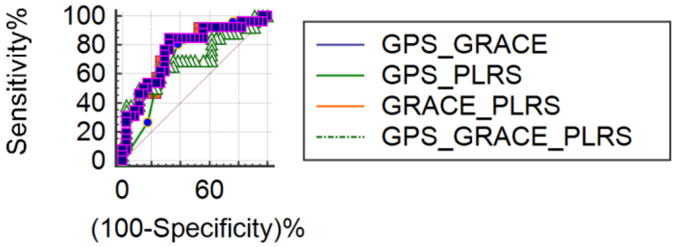

(3) Univariate ROC curve analysis in non-acute infection group.

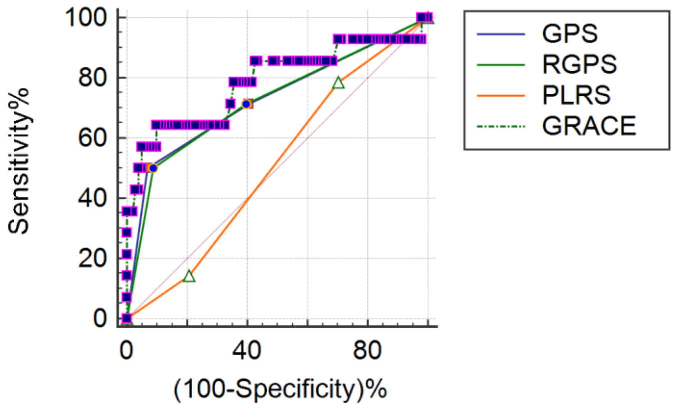

(4) Multivariate ROC curve analysis in non-acute infection group.

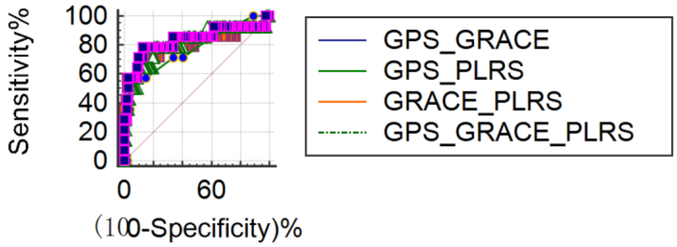

Supplementary Table 7. Subgroup results based on whether the patients had an acute infection or not.

(1) Pairwise comparison of univariate receiver operating characteristic (ROC) curves in acute infection group.

Variable	AUC	SE [a]	95% CI [b]
GPS	0.693	0.0619	0.560 to 0.806
RGPS	0.664	0.0636	0.530 to 0.781
PLRS	0.551	0.0720	0.417 to 0.680
GRACE	0.700	0.0707	0.568 to 0.812

GPS ~ RGPS	
Difference between areas	0.0288
Standard Error [a]	0.0245
95% Confidence Interval	-0.0192 to 0.0769
z statistic	1.177
Significance level	P = 0.2394
**GPS ~ PLRS**	
Difference between areas	0.142
Standard Error [a]	0.0901
95% Confidence Interval	-0.0347 to 0.319
z statistic	1.575
Significance level	P = 0.1153
**GPS ~ GRACE**	
Difference between areas	0.00735
Standard Error [a]	0.0793
95% Confidence Interval	-0.148 to 0.163
z statistic	0.0927
Significance level	P = 0.9261
**RGPS ~ PLRS**	
Difference between areas	0.113
Standard Error [a]	0.0909
95% Confidence Interval	-0.0650 to 0.291
z statistic	1.245

Significance level	P = 0.2133
RGPS ~ GRACE	
Difference between areas	0.0362
Standard Error [a]	0.0776
95% Confidence Interval	-0.116 to 0.188
z statistic	0.467
Significance level	P = 0.6408
PLRS ~ GRACE	
Difference between areas	0.149
Standard Error [a]	0.0915
95% Confidence Interval	-0.0300 to 0.329
z statistic	1.632
Significance level	P = 0.1027

(2) Pairwise comparison of multivariate ROC curves in acute infection group.

Variable	AUC	SE [a]	95% CI [b]
GPS_GRACE	0.762	0.0641	0.635 to 0.863
GPS_PLRS	0.701	0.0683	0.569 to 0.813
GRACE_PLRS	0.705	0.0711	0.573 to 0.816
GPS_GRACE_PLRS	0.761	0.0643	0.634 to 0.862

GPS_GRACE ~ GPS_PLRS	
Difference between areas	0.0611
Standard Error [a]	0.0398
95% Confidence Interval	-0.0170 to 0.139
z statistic	1.533
Significance level	P = 0.1252
GPS_GRACE ~ GRACE_PLRS	
Difference between areas	0.0577
Standard Error [a]	0.0511
95% Confidence Interval	-0.0425 to 0.158
z statistic	1.129
Significance level	P = 0.2589

GPS_GRACE ~ GPS_GRACE_PLRS	
Difference between areas	0.00113
Standard Error [a]	0.00699
95% Confidence Interval	-0.0126 to 0.0148
z statistic	0.162
Significance level	P = 0.8715
GPS_PLRS ~ GRACE_PLRS	
Difference between areas	0.00339
Standard Error [a]	0.0764
95% Confidence Interval	-0.146 to 0.153
z statistic	0.0444
Significance level	P = 0.9646
GPS_PLRS ~ GPS_GRACE_PLRS	
Difference between areas	0.0600
Standard Error [a]	0.0377
95% Confidence Interval	-0.0139 to 0.134
z statistic	1.590
Significance level	P = 0.1118
GRACE_PLRS ~ GPS_GRACE_PLRS	
Difference between areas	0.0566
Standard Error [a]	0.0498
95% Confidence Interval	-0.0410 to 0.154
z statistic	1.136
Significance level	P = 0.2559

(3) Comparisons between multivariate ROC curves with high scores and univariate ROC curves in acute infection group.

Variable	AUC	SE [a]	95% CI [b]
GPS_GRACE_PLRS	0.761	0.0643	0.634 to 0.862
GPS_GRACE	0.762	0.0641	0.635 to 0.863
GPS	0.693	0.0619	0.560 to 0.806
RGPS	0.664	0.0636	0.530 to 0.781
GRACE	0.700	0.0707	0.568 to 0.812

GPS_GRACE_PLRS ~ GPS	
Difference between areas	0.0684
Standard Error [a]	0.0378
95% Confidence Interval	-0.00562 to 0.143
z statistic	1.811
Significance level	P = 0.0701
GPS_GRACE_PLRS ~ RGPS	
Difference between areas	0.0973
Standard Error [a]	0.0413
95% Confidence Interval	0.0164 to 0.178
z statistic	2.357
Significance level	P = 0.0184
GPS_GRACE_PLRS ~ GRACE	
Difference between areas	0.0611
Standard Error [a]	0.0496
95% Confidence Interval	-0.0362 to 0.158
z statistic	1.231
Significance level	P = 0.2183
GPS_GRACE ~ GPS	
Difference between areas	0.0696
Standard Error [a]	0.0367
95% Confidence Interval	-0.00242 to 0.142
z statistic	1.894
Significance level	P = 0.0582
GPS_GRACE ~ RGPS	
Difference between areas	0.0984
Standard Error [a]	0.0407
95% Confidence Interval	0.0186 to 0.178
z statistic	2.418
Significance level	P = 0.0156
GPS_GRACE ~ GRACE	
Difference between areas	0.0622
Standard Error [a]	0.0501
95% Confidence Interval	-0.0360 to 0.160

z statistic	1.242
Significance level	P = 0.2143

(4) Pairwise comparison of univariate ROC curves in non-acute infection group.

Variable	AUC	SE [a]	95% CI [b]
GPS	0.731	0.0811	0.640 to 0.809
RGPS	0.726	0.0796	0.635 to 0.805
PLRS	0.510	0.0685	0.415 to 0.604
GRACE	0.786	0.0835	0.700 to 0.857

GPS ~ RGPS	
Difference between areas	0.00460
Standard Error [a]	0.00983
95% Confidence Interval	-0.0147 to 0.0239
z statistic	0.467
Significance level	P = 0.6402
**GPS ~ PLRS**	
Difference between areas	0.221
Standard Error [a]	0.0755
95% Confidence Interval	0.0730 to 0.369
z statistic	2.927
Significance level	P = 0.0034
**GPS ~ GRACE**	
Difference between areas	0.0552
Standard Error [a]	0.0750
95% Confidence Interval	-0.0919 to 0.202
z statistic	0.735
Significance level	P = 0.4621
**RGPS ~ PLRS**	
Difference between areas	0.216
Standard Error [a]	0.0742
95% Confidence Interval	0.0709 to 0.362
z statistic	2.915

Significance level	P = 0.0036
RGPS ~ GRACE	
Difference between areas	0.0598
Standard Error [a]	0.0743
95% Confidence Interval	-0.0859 to 0.205
z statistic	0.804
Significance level	P = 0.4213
PLRS ~ GRACE	
Difference between areas	0.276
Standard Error [a]	0.0823
95% Confidence Interval	0.115 to 0.438
z statistic	3.355
Significance level	P = 0.0008

(5) Pairwise comparison of multivariate ROC curves in non-acute infection group.

Variable	AUC	SE [a]	95% CI [b]
GPS_GRACE	0.802	0.0871	0.717 to 0.870
GPS_PLRS	0.760	0.0756	0.672 to 0.835
GRACE_PLRS	0.811	0.0778	0.728 to 0.878
GPS_GRACE_PLRS	0.832	0.0788	0.751 to 0.895

GPS_GRACE ~ GPS_PLRS	
Difference between areas	0.0414
Standard Error [a]	0.0630
95% Confidence Interval	-0.0822 to 0.165
z statistic	0.656
Significance level	P = 0.5117
GPS_GRACE ~ GRACE_PLRS	
Difference between areas	0.00955
Standard Error [a]	0.0381
95% Confidence Interval	-0.0652 to 0.0843
z statistic	0.250
Significance level	P = 0.8023

GPS_GRACE ~ GPS_GRACE_PLRS	
Difference between areas	0.0304
Standard Error [a]	0.0190
95% Confidence Interval	-0.00676 to 0.0676
z statistic	1.603
Significance level	P = 0.1088
**GPS_PLRS ~ GRACE_PLRS**	
Difference between areas	0.0509
Standard Error [a]	0.0757
95% Confidence Interval	-0.0975 to 0.199
z statistic	0.672
Significance level	P = 0.5014
**GPS_PLRS ~ GPS_GRACE_PLRS**	
Difference between areas	0.0718
Standard Error [a]	0.0574
95% Confidence Interval	-0.0407 to 0.184
z statistic	1.251
Significance level	P = 0.2110
**GRACE_PLRS ~ GPS_GRACE_PLRS**	
Difference between areas	0.0209
Standard Error [a]	0.0298
95% Confidence Interval	-0.0375 to 0.0793
z statistic	0.700
Significance level	P = 0.4837

(6) Comparisons between multivariate ROC curves with high scores and univariate ROC curves in non-acute infection group.

Variable	AUC	SE [a]	95% CI [b]
GPS_GRACE_PLRS	0.832	0.0788	0.751 to 0.895
GPS	0.731	0.0811	0.640 to 0.809
RGPS	0.726	0.0796	0.635 to 0.805
GRACE	0.786	0.0835	0.700 to 0.857

GPS_GRACE_PLRS ~ GPS	
Difference between areas	0.101
Standard Error [a]	0.0569
95% Confidence Interval	-0.0104 to 0.213
z statistic	1.777
Significance level	P = 0.0756
**GPS_GRACE_PLRS ~ RGPS**	
Difference between areas	0.106
Standard Error [a]	0.0566
95% Confidence Interval	-0.00525 to 0.217
z statistic	1.867
Significance level	P = 0.0619
**GPS_GRACE_PLRS ~ GRACE**	
Difference between areas	0.0460
Standard Error [a]	0.0314
95% Confidence Interval	-0.0155 to 0.107
z statistic	1.466
Significance level	P = 0.1426

Supplementary Figure 5. Receiver operating characteristic of three PLRSs.

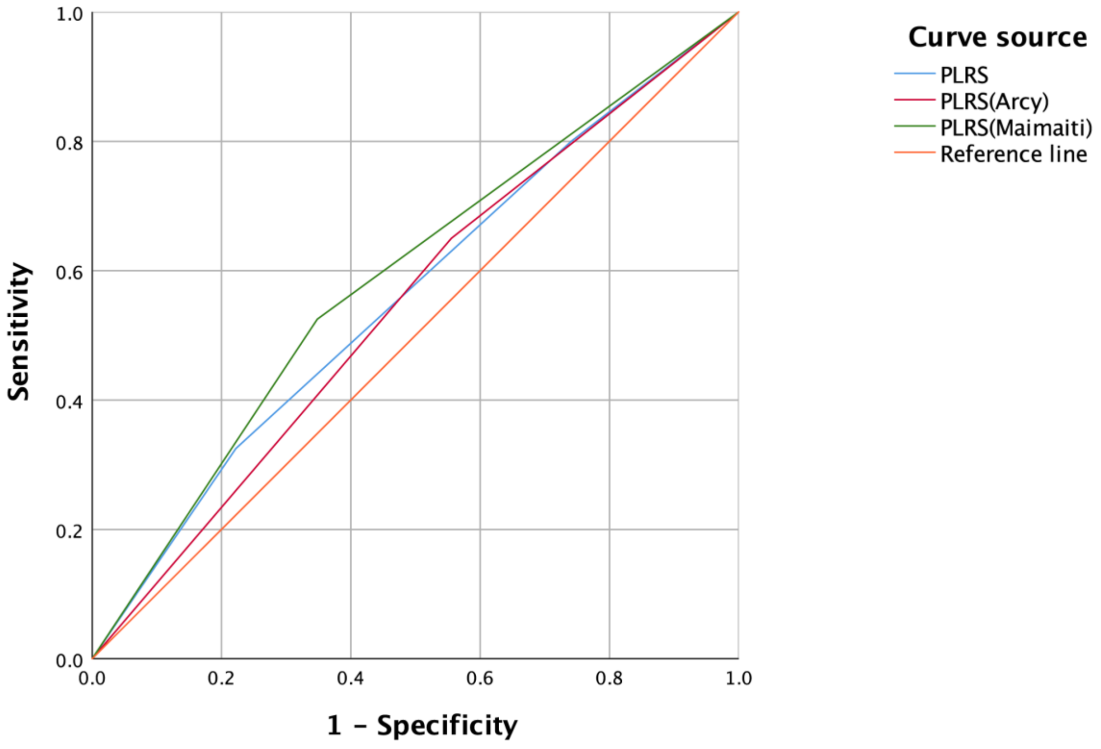

Supplementary Table 8. Receiver operating characteristic of three PLRSs.

Vairable	AUC	CI	P
PLRS	0.561	0.458-0.664	0.241
[a]PLRS(Arcy)	0.547	0.447-0.648	0.365
[b]PLRS(Maimaiti)	0.588	0.487-0.690	0.090

AUC = area under the curve; CI = confidence interval; PLRS = platelet-to-lymphocyte ratio score; [a]PLRS(Arcy) is defined as high PLRS (>137) and low PLRS (<137) from studies by Ayca et al. [b]PLRS(Maimaiti) is defined as high PLRS (>165.33) and low PLRS (<165.33) from studies by Maimaiti et al.
